# The optimal timing for intervention in patients with ST-segment elevation myocardial infarction and multivessel disease: a systematic review and meta-analysis

**DOI:** 10.3389/fcvm.2024.1389017

**Published:** 2024-08-09

**Authors:** Yi Chen, Meng Li, Yanqing Wu

**Affiliations:** The Second Affiliated Hospital, Jiangxi Medical College, Nanchang University, Nanchang, China

**Keywords:** ST-segment elevation myocardial infarction, multivessel disease, percutaneous coronary intervention, immediate multivessel PCI, staged multivessel PCI

## Abstract

**Purpose:**

The optimal timing for nonculprit vascular reconstruction surgery in patients with ST-segment elevation myocardial infarction (STEMI) and multivessel coronary disease (MVD) is still controversial. Our aim was to explore the optimal intervention time for percutaneous coronary intervention (PCI) in STEMI patients who underwent MVD.

**Methods:**

The PubMed/Medline, EMBASE, Cochrane Library, and ClinicalTrials.gov databases were searched from inception to January 1, 2024 for clinical studies comparing immediate multivessel PCI and staged multivessel PCI in patients with STEMI. The primary outcomes were death from any cause, cardiovascular death, noncardiac death, myocardial infarction (MI) and unplanned ischemia-driven revascularization. The secondary outcomes were ischemic stroke, stent thrombosis, renal dysfunction and major bleeding. The risk ratios (RRs) and odds ratios (ORs) were calculated with fixed-effects models and random-effects models, and 95% confidence intervals (CIs) were calculated.

**Findings:**

Five randomized trials with 2,782 patients and six prospective observational studies with 3,131 patients were selected for inclusion in this meta-analysis. The staged PCI group had significantly lower pooled RRs for myocardial infarction (0.43, 95% *CI *= 0.27–0.67; *P* = 0.0002) and unplanned ischemia-driven revascularization (0.57, 95% *CI *= 0.41–0.78; *P* = 0.0004). There were no significant differences in any cause of death, cardiovascular cause of death, or noncardiac cause of death. However, the results of prospective observational studies in the real world indicated that the staged PCI group had significantly lower pooled ORs for all-cause mortality (2.30, 95% *CI* = 1.22–4.34; *P* = 0.01), cardiovascular death (2.29, 95% *CI* = 1.10–4.77; *P* = 0.03), and noncardiovascular death (3.46, 95% *CI* = 1.40–8.56; *P* = 0.007).

**Implications:**

According to our randomized trial analysis, staged multivessel PCI significantly reduces the risk of myocardial infarction and unplanned ischemia-driven revascularization compared to immediate multivessel PCI. There was no significant difference between the two groups in terms of all-cause mortality, cardiovascular mortality, or noncardiovascular mortality risk. However, prospective non-randomized studies suggest there might be a benefit in mortality in the staged PCI group. Therefore, staged multivessel PCI may be the optimal PCI strategy for STEMI patients with MVD.

## Introduction

Multiple coronary artery disease (CAD) is present in most patients with acute ST-segment elevation myocardial infarction (STEMI) and is associated with an increased risk of myocardial infarction recurrence and death (1–5). Primary percutaneous coronary intervention (PCI) is the preferred strategy for restoring culprit artery blood flow in STEMI patients (6–8). For decades, multiple trials have compared the advantages of pure culprit vascular PCI and complete percutaneous revascularization, thus demonstrating the advantages of complete percutaneous revascularization. An analysis in the COMPLETE trial showed that the benefits of complete percutaneous revascularization are consistent with those of PCI with only the culprit lesion (9). The Preventive Angioplasty in Acute Myocardial Infarction (PRAMI) trial reported that immediate complete angioplasty reduced the incidence of cardiovascular events compared to PCI limited to the culprit lesion (10). The Complete vs. Lesion Only Primary PCI Trial (CvL PRIT) showed that the incidence of cardiovascular composite outcomes [major adverse cardiovascular events (MACEs)] caused by complete percutaneous revascularization during hospitalization was lower than that caused by simple infarct-related artery treatment (11). The Third Danish Study of Optimal Act Treatment of Patients with ST Segment Elevation Myocardial Infarction - Primary PCI in Multivessel Disease (DANAMI-3 PRIMULTI) initiative also reported of significant benefits in terms of hemodynamic stability in STEMI and multivessel coronary disease (MVD) patients treated with multivessel PCI (12). The limitations of these trials include the fact that whole-blood vessel reconstruction surgery was performed in stages and did not address the optimal timing for nonculprit lesion blood vessel reconstruction; moreover, comparisons between immediate PCI and staged PCI are lacking. In similar situations, some physicians tend to prefer immediate vascular reconstruction for nonculprit lesions, whereas others believe that a phased vascular reconstruction strategy is more beneficial.

Hence, we conducted this meta-analysis to examine the advantages and disadvantages of immediate multivessel PCI and staged multivessel PCI for STEMI patients with multivessel lesions.

## Methods

### Literature search and selection

We searched PubMed/Medline, Embase, the Cochrane Library, and ClinicalTrials.gov from inception through April 1, 2024. We searched for studies with medical search terms and related variants, including “coronary artery disease” or “disease, coronary artery” or “coronary artery disease” or “coronary heart disease” or “multivessel coronary artery disease” or “myocardial infarction” or “cardiovascular stroke” or “myocardial infarction” and “immediate PCI” and “staged PCI”. We searched for randomized controlled trials (RCTs) by using search filters from McMaster University. We also searched the corresponding references of each retrieved study to identify additional studies. All of the search results were evaluated according to the Preferred Reporting Items for Systematic Reviews and Meta-Analyses (PRISMA) statement (13).

The efficacy and safety of immediate and staged PCI between hemodynamically stable STEMI or non-STEMI patients and MVD patients were compared in all of the studies. The inclusion criteria for this study were as follows: (i) a clinical study using different strategies for multivessel revascularization, (ii) a clinical environment in which revascularization was performed for acute coronary syndrome (ACS)-STEMI or non-STEMI, and (iii) a clinical study analyzing the primary efficacy outcomes, including all-cause death, cardiovascular death, myocardial infarction, unplanned ischemia-driven revascularization, and major adverse events, including ischemic stroke, stent thrombosis, renal dysfunction and major bleeding. The exclusion criteria were as follows: (i) duplicate papers related to the same experiment; (ii) system evaluations, comments, case reports, meetings, editorials, or noncomparative studies; and (iii) clinical studies that did not report the required results.

### Data extraction and quality assessment

The data extraction and quality evaluation were independently conducted by two researchers (C.Y. and M.L.). The data included baseline characteristics, intervention measures, comparisons, sample sizes, and follow-up times. The results included death from any cause, cardiovascular death, myocardial infarction, unplanned ischemia-driven revascularization, ischemic stroke, stent thrombosis, renal insufficiency and major bleeding.

The methodological quality of the 11 included clinical studies was assessed by using the Cochrane Collaboration risk of bias tool (Review Manager 5.3), which included the following sections: selection, performance, detection, attrition, and reporting. The two investigators cross-checked the data. Any disagreements were resolved by another investigator (W. Y. Q.).

## Statistical analysis

The statistical analyses were performed by using Review Manager Version 5.3.3 (Copenhagen: The Nordic Cochrane Centre, The Cochrane Collaboration, 2014). Efficacy and safety were measured as dichotomous outcome variables and compared between the immediate PCI group and the staged PCI group. The pooled odds ratio (OR) and the corresponding 95% confidence interval (*CI*) were calculated for the comparative analyses. We assessed heterogeneity by using the *I*^2^ test and Cochran's *χ*^2^ test. The total variation in the studies was described by using the *I*^2^ statistic, which reflects heterogeneity. An *I*^2^ ≥ 50% or a corresponding *P* < 0.10 indicated significant heterogeneity among the different studies. When *I*^2^ was <50% and *P* was >0.10, we reported the results of fixed-effects models as sensitivity analyses. All of the *P*-values were two-tailed, with statistical significance specified at 0.05 and *CI*s reported at the 95% level. When *I*^2^ was >50%, a sensitivity analysis was further performed by sequentially deleting each study and reanalyzing the datasets of all of the remaining studies.

## Results

### Study selection and quality assessment

**Figure 1:**
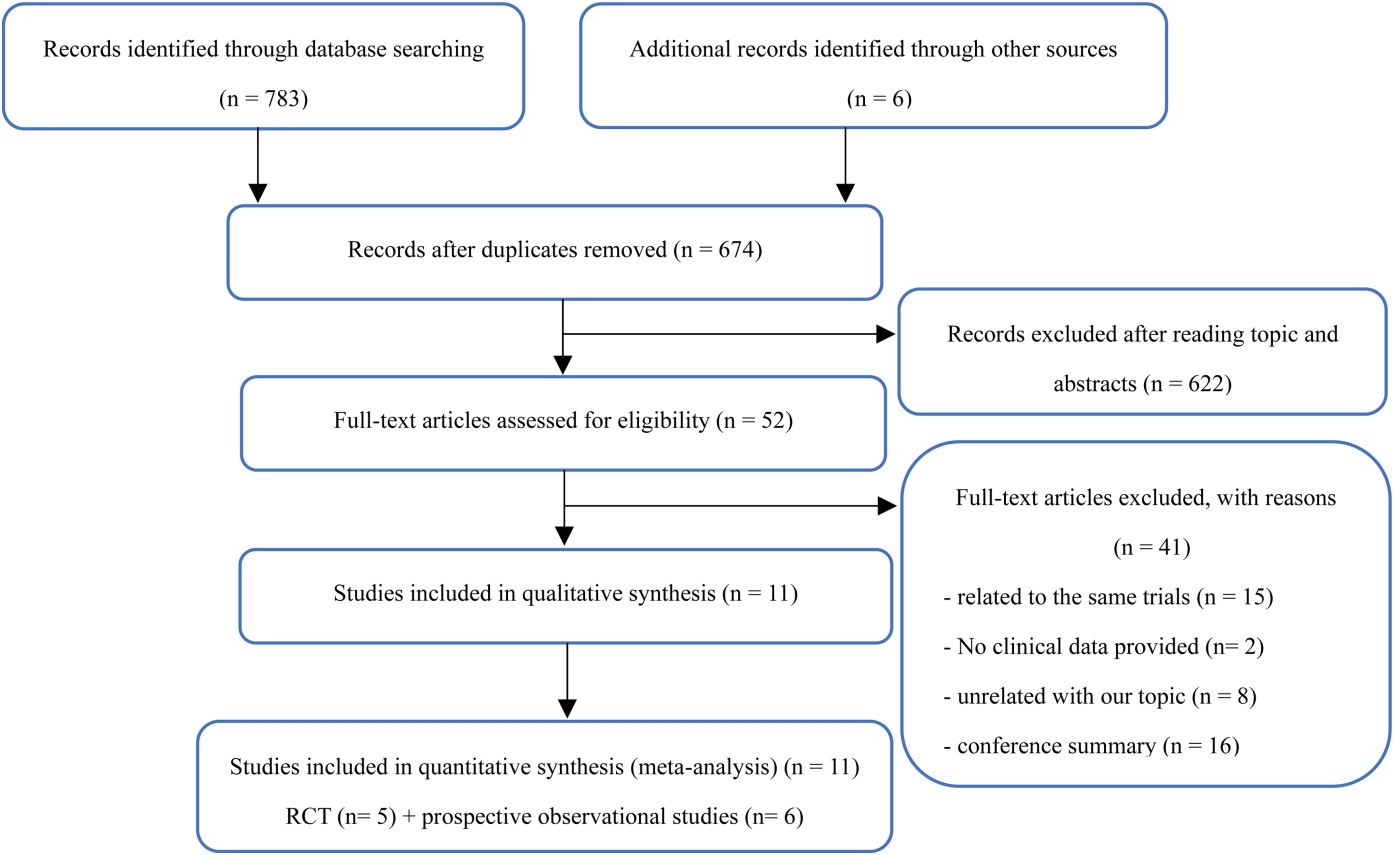
Study search diagram adapted from the preferred reporting items for systematic reviews and meta-analyses (PRISMA) statement.

The research selection flowchart is shown in Figure 1. According to the abovementioned search strategy, 674 citations were obtained after removing duplicate records from the online database from January 1, 2000, to January 1, 2021. The full texts of 52 articles were reviewed in detail, and 41 articles were further excluded because they were related to the same trials (*n* = 15), lacked clinical data (*n* = 2), had unrelated topics (*n* = 8), or were conference abstracts (*n* = 16). Finally, 5 randomized trials with 2,782 patients and 6 prospective observational studies with 3,131 patients were selected for inclusion in this meta-analysis (14–24).

The characteristics, quality evaluation results, and demographic information of the included studies are summarized in Table 1 and Supplementary Table S1. The follow-up durations ranged from 1 year to 3 years, and the sample sizes of the trials ranged from 78 to 1,525 patients. Moreover, the risk of bias was assessed in five studies and was generally found to be low in each study (Supplementary Figure S1).

**Table 1:** Baseline characteristics of included studies.

Study	Stahli 2023 (MULTISTARS AMI)	Park 2023 (COCUA)	Diletti 2023 (BIOVASC)	Kim 2021	Ahn 2020 (KAMIR-NIH)	Kim 2017	Tarasov 2017	Chung 2016	Kornowski 2011 (HORIZONS-AMI)	Maamoun 2011	Politi 2010
Number of patients (Immediate PCI/Staged PCI)	840 (418/422)	209 (103/106)	1,525 (764/761)	861 (351/510)	606 (254/352)	753 (316/437)	136 (67/69)	107 (66/41)	668 (275/393)	78 (42/36)	130 (65/65)
Timing of staged PCI	37 (30–43) days	4.4 ± 7.0 days	15 (4–28) days	5 (3–6) days	NA	NA	10.1 ± 5.1 days	NA	30 (6–51) days	<7 days	56.8 days
Follow-up	1 year	1 year	1 year	3 years	1 year	3.4 years	1 year	1 year	1 year	1 year	2.5 years
Median age (year)	66/64	63.3/62.2	65.7/65.3	62.3/61.9	62/62	62.3/63.5	58.6/59.1	64/62	62/63.5	54.5/52.3	64.5/64.1
Sex (male%)	76.8/80.8	79.6/83	78.3/77.4	81.5/81.6	80.7/79.8	76.3/71.8	71.6/62.3	68.2/78	79.6/80.9	95.2/88.9	76.9/80
LVEF (%)	NA	50.5/52.2	NA	49.3/51.2	48/52	52.4/53.3	50.7/51.8	48/49	57.4/58.6	45.5/45	45.4/45.9
Hypertension (%)	54.5/50.2	54.3/45.2	55.4/51.9	50.7/47.6	48.8/44.3	49.4/52.4	94/88.4	NA	54.9/57.5	38.1/33.3	49.2/64.6
Diabetes (%)	15.8/15.4	40.7/34.9	20.7/21.4	30.5/26.7	30.7/24.1	36.1/31.6	23.9/20.3	NA	15.3/18.1	40.5/55.6	13.8/18.5
Dyslipidemia (%)	26.8/27.1	36.8/38.6	50.5/52.5	10/10.6	8.3/12.2	28/32	NA	NA	48/41.7	57.1/44.4	NA
Smoke (%)	33.9/35.4	36.8/41.5	33.4/31.7	47/44.1	48/44.6	63.9/62.9	NA	NA	61.3/62.8	52.4/55.6	NA
No. of vessels with relevant nonculprit lesions ≥2 (%)	NA	16.5/20.8	16/20.8	33/39.4	88.2/96	38.6/42.1	47.8/44.9	NA	NA	26.2/22.2	29.2/44.6
Volume of contrast material (Index procedure) (ml)	250/170	NA	200/140	NA	NA	NA	325.8/373	NA	275/235	NA	NA
Hospital day (Index procedure) (days)	4/4	10.6/9	3/4	4/6	4/6	NA	NA	NA	NA	NA	4.8/5.4

### Primary efficacy outcomes

#### Death

**Figure 2:**
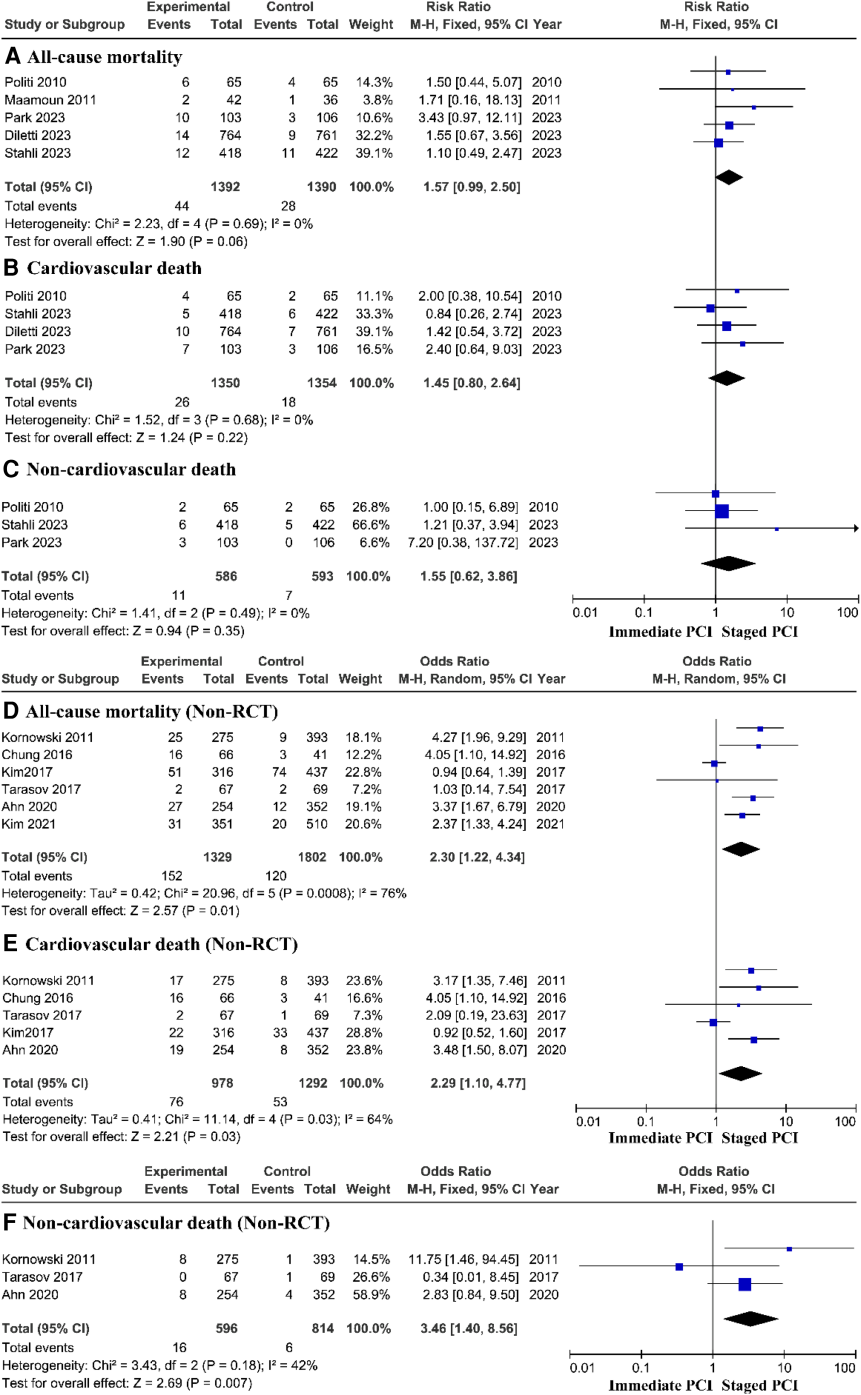
Mortality rate based on RCTs and real-world prospective studies.

To evaluate the main outcome, the meta-analysis included 11 trials. The estimated main therapeutic outcomes for all causes of death, cardiovascular causes of death, and noncardiac causes of death are shown in Figure 2. Research statistics based on RCTs have shown that there is no significant difference in the risk of all-cause mortality, cardiovascular mortality, or noncardiovascular mortality between immediate PCI and staged PCI. However, prospective research analysis based on the real world showed that the immediate PCI group had greater all-cause mortality, with a pooled *OR* of 2.30 (95% *CI* = 1.22–4.34, *P* = 0.01; *P* = 0.0008 for heterogeneity; *I*^2^ = 76%) (Figure 2D); cardiovascular mortality, with a pooled *OR* of 2.29 (95% *CI* = 1.10–4.77, *P* = 0.03; *P* = 0.03 for heterogeneity; *I*^2^ = 64%) (Figure 2E); and noncardiovascular mortality, with a pooled *OR* of 3.46 (95% *CI* = 1.40–8.56, *P* = 0.007; *P* = 0.18 for heterogeneity; *I*^2^ = 42%) (Figure 2F), than did the staged PCI group.

#### Myocardial infarction

**Figure 3:**
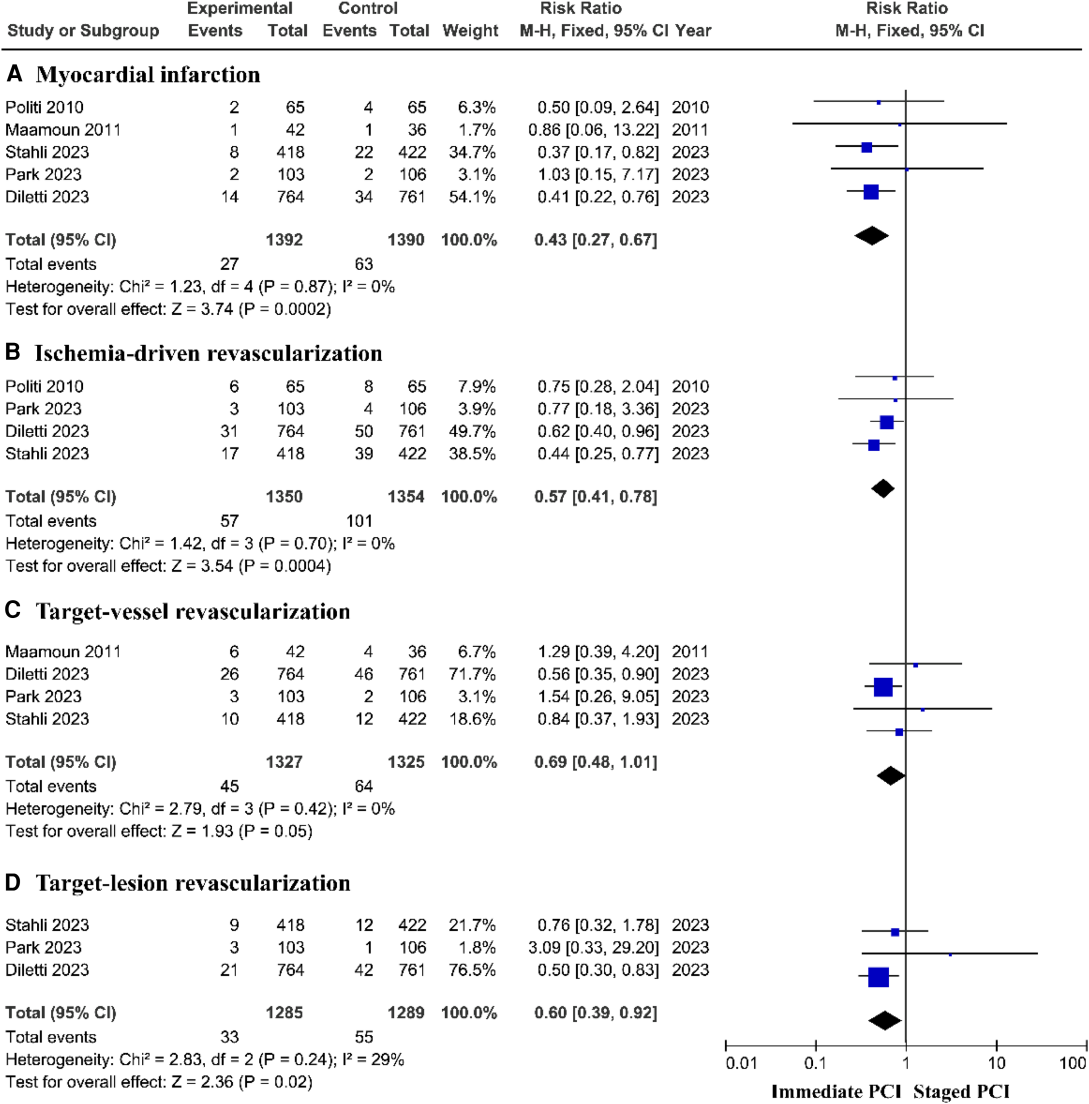
The incidence of myocardial infarction and ischemia-driven revascularization based on RCTs.

The composite outcome of myocardial infarction showed that the staged PCI group had a lower incidence of myocardial infarction than did the immediate PCI group, with a pooled *RR* of 0.43 (95% *CI *= 0.27–0.67, *P* = 0.0002; *P* = 0.87 for heterogeneity; *I*^2^ = 0%) (Figure 3A). A prospective controlled study based on the real world showed no significant difference in the risk of myocardial infarction between immediate PCI and staged PCI (Supplementary Figure S2A).

#### Ischemia-driven revascularization

The risk of unplanned ischemia-driven revascularization was greatly reduced among patients treated with staged PCI, with a pooled *RR* of 0.57 (95% *CI *= 0.41–0.78, *P* = 0.0004; *P* = 0.70 for heterogeneity; *I*^2^ = 0%) (Figure 3B) and target lesion revascularization (TLR), with a pooled *RR* of 0.60 (95% *CI *= 0.39–0.92, *P* = 0.02; *P* = 0.24 for heterogeneity; *I*^2^ = 29%); moreover, there was no significant difference in the incidence of target vessel revascularization (TVR) between the immediate PCI group and the staged PCI group (Figures 3C, D). However, prospective controlled studies based on the real world showed no significant difference in the risk of unplanned ischemic revascularization between immediate PCI and staged PCI (Supplementary Figures S2B–D).

#### Subgroup analysis

**Figure 4:**
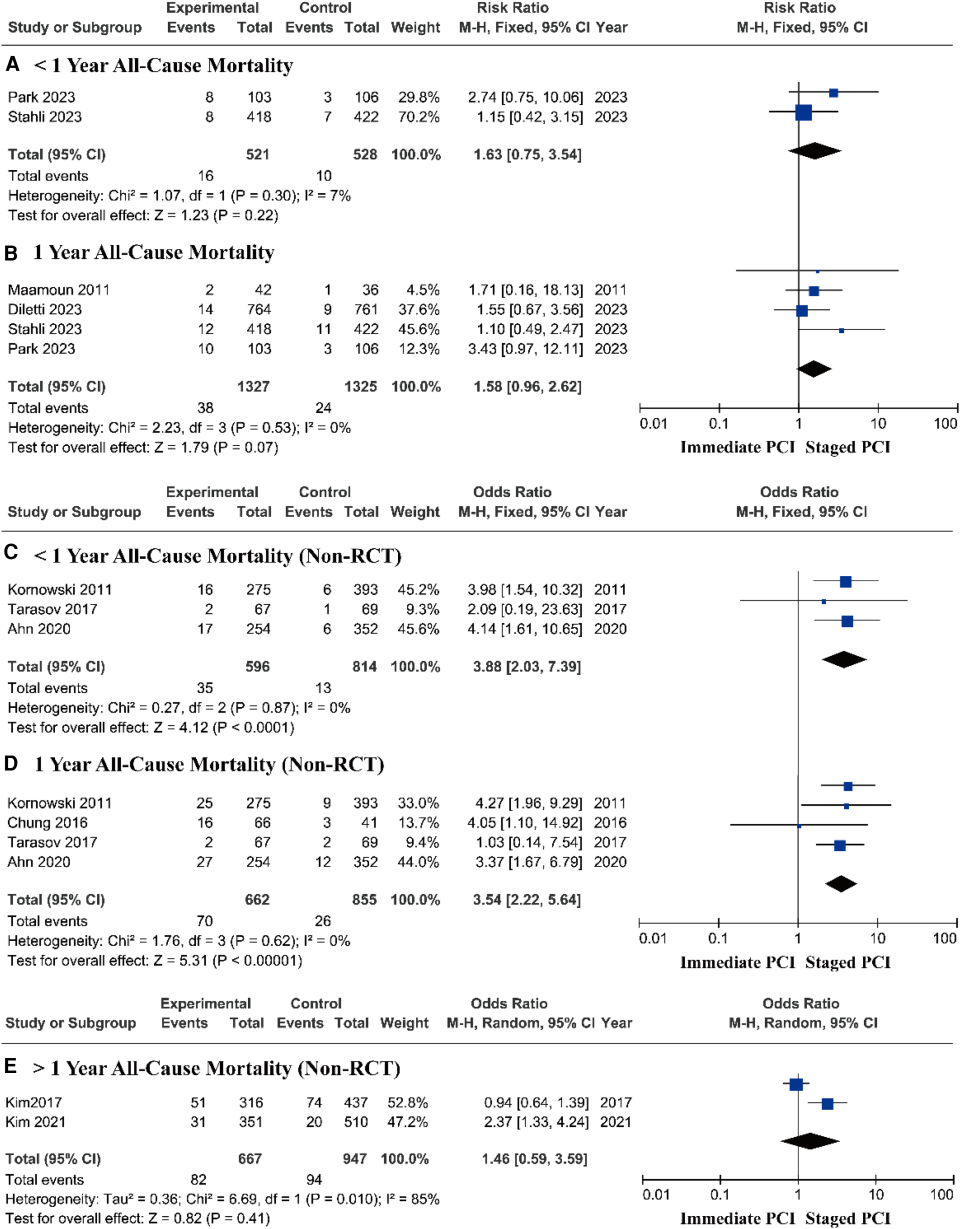
Short- and long-term all-cause mortality based on RCTs and real-world prospective studies.

Research based on RCTs showed that there was no significant difference in the risk of all-cause mortality between immediate PCI and staged PCI during follow-up periods of less than 1 year and at 1 year, respectively (Figures 4A,B). Therefore, we focused on prospective research based on the real world, in which all-cause mortality, with a pooled *OR* of 3.88 (95% *CI* = 2.03–7.39, *P* < 0.0001; *P* = 0.87 for heterogeneity; *I*^2^ = 0%) during short-term follow-up (Figure 4C) and a pooled *OR* of 3.54 (95% *CI* = 2.22–5.64, *P* < 0.00001; *P* = 0.62 for heterogeneity; *I*^2^ = 0%) during the one-year follow-up in the staged PCI group were lower than those in the immediate PCI group (Figure 4D). After more than one year of follow-up, there was no significant difference in the risk of all-cause mortality between immediate PCI and staged PCI (Figure 4E).

**Figure 5:**
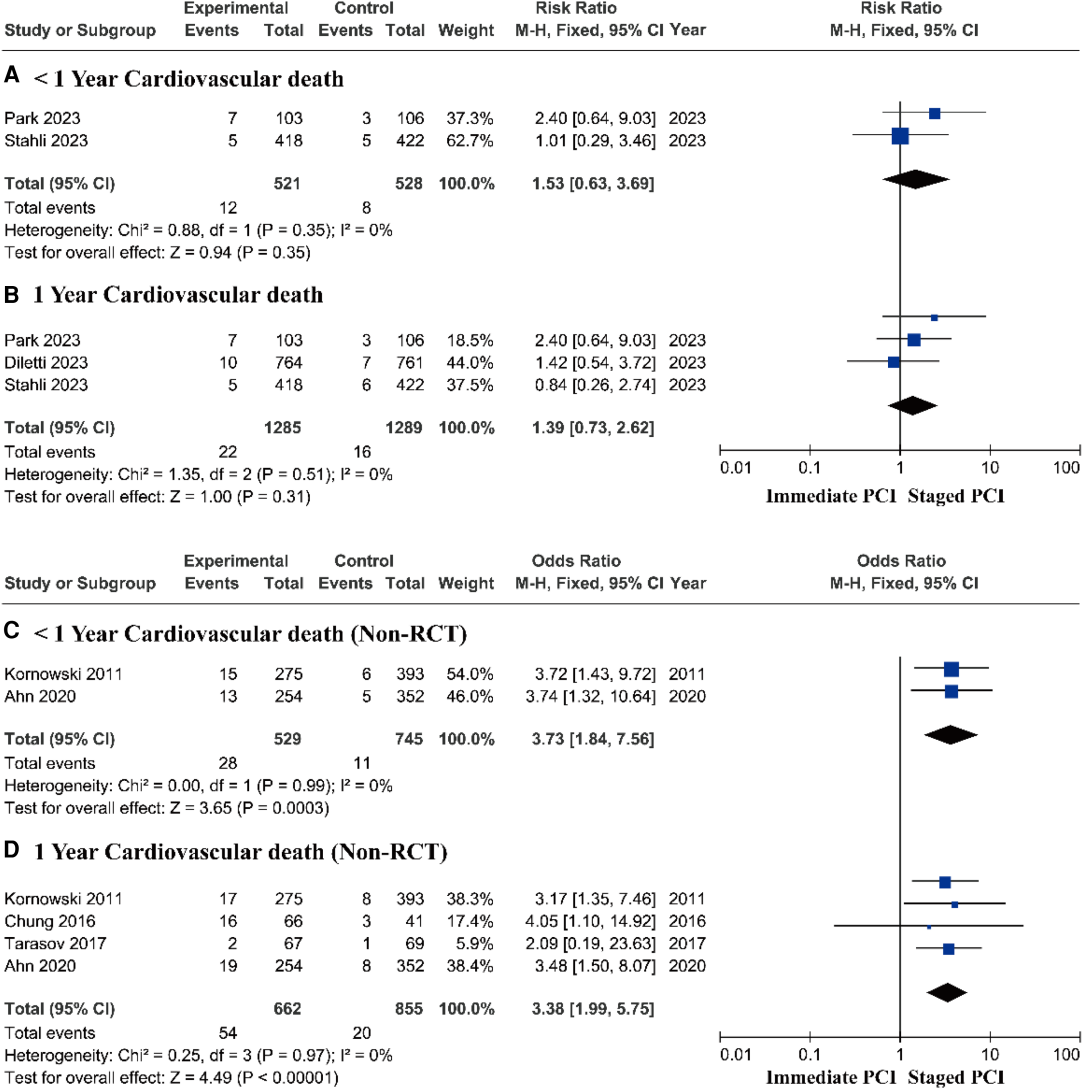
Short- and long-term cardiovascular mortality based on RCTs and real-world prospective studies.

Research based on RCTs showed that there was no significant difference in the risk of cardiovascular death between immediate PCI and staged PCI (Figures 5A,B). However, prospective research analysis based on real-world data demonstrated that staged PCI still had advantages in terms of cardiovascular mortality, with a pooled *OR* of 3.73 (95% *CI* = 1.84–7.56, *P* = 0.0003; *P* = 0.99 for heterogeneity; *I*^2^ = 0%) during short-term follow-up (Figure 5C) and a pooled *OR* of 3.38 (95% *CI* = 1.99–5.75, *P* < 0.00001; *P* = 0.97 for heterogeneity; *I*^2^ = 0%) during one-year follow-up (Figure 5D).

#### Safety outcomes

**Figure 6:**
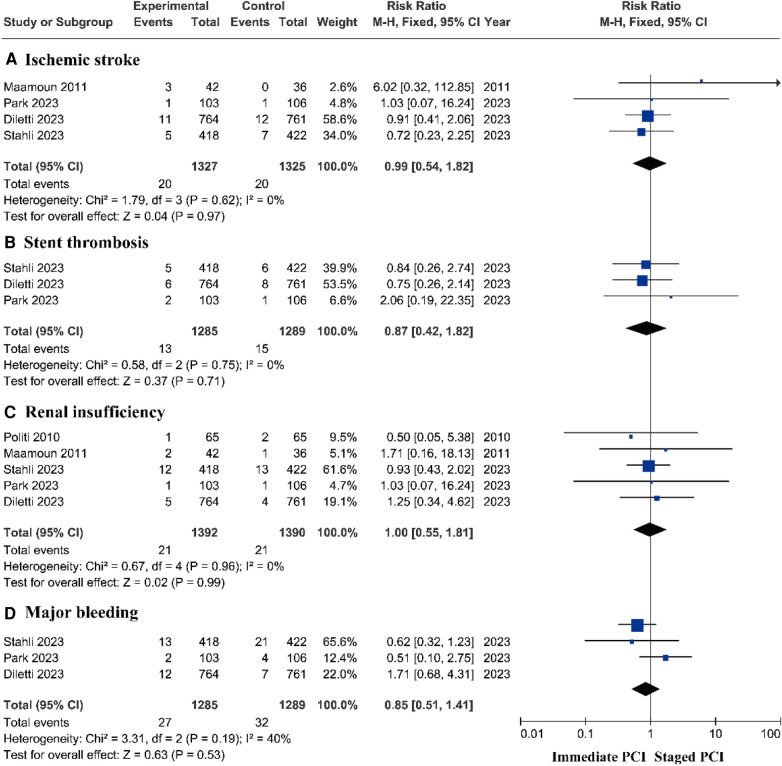
Adverse outcomes based on RCTs.

There was no significant difference in the risk of ischemic stroke (14–16, 18, 22, 23), stent thrombosis (14–16, 18–20, 23), renal insufficiency (14–16, 22, 24), or major bleeding (14–16, 23) between the immediate PCI group and the staged PCI group (Figures 6A–D and Supplementary Figure S3).

## Discussion

Most of the studies that were included in this meta-analysis were multicenter, randomized, actively controlled trials with a low risk of bias. All of the included clinical studies included STEMI and MVD patients. This study is the first to systematically analyze the clinical outcomes of immediate and staged PCI strategies for multivessel lesions and the first to compare the short-term and long-term outcomes of immediate and staged PCI. We also added three large-scale RCTs that were newly published in the previous year (14–16). These data indicated that staged PCI was superior to immediate PCI in reducing the risk of outcomes such as all-cause mortality, cardiovascular mortality, noncardiovascular mortality, myocardial infarction, and ischemia-driven coronary revascularization; however, there were no significant differences in ischemic stroke, stent thrombosis, renal function injury, or major bleeding. In addition, the incidence of all-cause and cardiovascular mortality within 1 year of staged PCI was lower than that of immediate PCI, but there seemed to be no significant difference between the two results for follow-up beyond 1 year.

The main analysis of this study showed that, in RCT-based studies, there was no significant difference in all-cause mortality, cardiovascular mortality, or noncardiovascular mortality between the immediate PCI group and the staged PCI group. However, real-world prospective observational studies have shown that staged PCI significantly reduces the risk of all-cause mortality, cardiovascular mortality, and noncardiovascular mortality. However, we observed significant heterogeneity in the statistical results for all-cause mortality and cardiovascular mortality in real-world prospective observational studies (*I*^2 ^> 50%), and further sensitivity analysis demonstrated that Kim et al.'s study reported of opposing results to our meta-analysis (20). We believe that the reason for this bias is that the patients in the staged PCI group were divided into two groups based on the 1-week time course, and there was no statistically significant difference in all-cause mortality or cardiovascular death between immediate PCI and staged PCI within 1 week. However, this did not affect the final outcome of our statistical analysis. We cannot deny that another important reason for the higher mortality rate of immediate PCI in nonrandomized real-world studies is selection bias. Operators tend to perform PCI of the non-culprit lesion when the lesion is perceived to be high risk or when there's hemodynamic instability (such as cardiogenic shock). On the other hand, they tend to stage PCI of the non-culprit lesion when the lesion and/or patient is stable. In addition, we conducted a subgroup analysis based on the different follow-up times for immediate PCI and staged PCI. We found that there was no significant difference in the risk of all-cause or cardiovascular death between immediate PCI patients and staged PCI patients during follow-up periods of 1 month and 1 year (Stahli's shortest follow-up time was 6 months; these patients were also included in the short-term follow-up group (14). However, prospective observational studies in the real world have shown that patients with staged PCI exhibit a greater survival rate than patients with immediate PCI during follow-up periods of one month and one year. This effect could be due to the fact that, in the RCTs, patients were strictly selected, and high-risk conditions such as cardiogenic shock, coronary artery bypass grafting, and chronic total occlusion of the coronary artery were excluded from the five included studies. However, prospective studies in the real world lack rigor in patient selection, which inevitably leads to bias. Caution is recommended when extrapolating our research results to real-world populations. In cases in which the follow-up time exceeded 1 year, we did not collect any data related to the RCT; therefore, we examined prospective observational studies in the real world to seek answers. We found no significant difference in the risk of all-cause mortality over 1 year between immediate PCI and staged PCI and included two studies on cardiovascular mortality (a 14-year-old RCT with a small base and a prospective observational study from the real world), which we were unable to combine. Moreover, these two studies seem to have reported opposite results for cardiovascular mortality. In recent years, the follow-up time of randomized controlled trials has been approximately one year, and follow-up times exceeding one year are rare. The follow-up results of our subgroup analysis over one year were all retrospective, and additional randomized controlled trials are needed in the future to obtain more valuable results. However, it is undeniable that over time, there are more factors affecting the survival rates of patients, including hypertension, diabetes and other diseases, as well as drug use and different lifestyle habits.

Two large-scale RCTs that were published in the previous year (the MULTISTARS AMI trial (14) and BIOVASC (16) trial) showed that the risk of cardiovascular composite outcomes was not lower among patients who underwent immediate multivessel PCI than among those who underwent staged multivessel PCI. The intergroup differences in the primary endpoint were caused by the decreased risk of nonfatal myocardial infarction and early unplanned ischemia-driven revascularization, which was possibly due to the fact that patients in the staged PCI group were more likely to be recommended for early ischemia-driven interventions when the coronary artery anatomy was known. Second, due to the fact that unplanned revascularization is defined as unplanned ischemia-driven revascularization performed due to symptoms of angina, ischemic changes on electrocardiography (ECG), or reversible signs of myocardial ischemia on noninvasive imaging, this may reduce the risk of overestimating the incidence of events. Another reason is that due to diffuse coronary artery vasoconstriction and systemic endothelial dysfunction, the severity of nonculprit vascular lesions may be overestimated during the first PCI. In addition, patients may be prone to plaque rupture and the development of acute coronary syndrome during the time window between the index date and staged surgery, thereby increasing the risk of unplanned ischemia-driven revascularization (25, 26). Moreover, most of the included studies indicated that the indications for PCI for nonculprit lesions in STEMI patients are mainly based on visual evaluation via coronary angiography, with low utilization rates of functional and imaging evaluations. The use of invasive imaging and coronary artery physiological examination may provide more comprehensive and accurate diagnostic grouping identification. Notably, after excluding surgery-related myocardial infarction (type 4) from the analysis, the abovementioned two studies showed that in patients who underwent staged PCI, the increased risk of myocardial infarction was not due to surgery-related events but rather due to spontaneous myocardial infarction (14, 16). Another notable issue is that, in Kim et al.'s study, there was a significant difference in the use of radial access between staged PCI and single-stage PCI (17). Unfortunately, we could not confirm the differences caused by the radial or femoral artery approach in the included studies. Moreover, although most of the included studies included MACEs, their definitions of MACEs were not the same. Stahli et al. added hospitalization for stroke and heart failure to the composite primary endpoint; although the incidence of these two outcomes was relatively low and there seemed to be no difference between the experimental groups, it cannot be denied that this may have led to bias toward noninferiority (14). Some studies included comprehensive ischemia-driven revascularization as a MACE, whereas others included target vessel revascularization as a MACE. Second, the incidence of ischemic stroke as a MACE varied across studies. For the abovementioned reasons, we did not evaluate the occurrence of MACEs during immediate PCI or staged PCI.

In theory, early complete revascularization of nonculprit blood vessels can reduce the risk of repeated revascularization, restore myocardial perfusion, and improve clinical outcomes, although immediate PCI may elicit risks such as prolonged surgical time and the use of contrast agents (27). Interestingly, due to the multiple rounds of revascularization involved in staged PCI, additional contrast agent doses are used, and the radiation exposure time is longer. Patients may have poor tolerance of contrast agents, thus resulting in a greater probability of renal function damage. However, based on the collected data, it appears that the incidence of renal injury among immediate PCI patients was greater than that among staged PCI patients, although this difference was not statistically significant. Of course, few studies have recorded the amount of contrast agent that was used, and there are many factors that affect kidney injury; moreover, we cannot attribute this solely to the use of contrast agents.

Our study did not demonstrate any difference in the risk of stent thrombosis or major bleeding between immediate PCI and staged PCI. The studies that we included were first conducted in 2010 and used bare metal stents (24). However, many myocardial infarction patients now use other types of stents, including drug-eluting stents and biodegradable stents, and the drugs that are used for postoperative antiplatelet therapy also differ (28–32). Therefore, we believe that the statistical results on stent thrombosis and major bleeding that were recorded in these studies alone are still insufficient, and additional research is needed in the future to document the risk of adverse reactions after immediate and staged PCI.

Another noteworthy point is that the average total hospitalization time of patients in the immediate PCI group was lower than that of patients in the staged PCI group, which may be attributed to the strategy of immediate multivessel PCI avoiding additional arterial puncture, staged revascularization, or a second hospitalization. In addition, due to the shorter hospital stay and lower incidence of revascularization, immediate complete revascularization may have potential economic value.

Our research had several potential limitations. First, to analyze the differences in short-term and long-term benefits between immediate PCI and staged PCI, we included observational and retrospective registration data and thereby could not eliminate the risk of confounding bias. Second, most of those studies included both male and Caucasian populations; therefore, our analysis results may not be applicable to different demographic environments. Third, our meta-analysis was not registered with PROSPERO. Fourth, most of the patients with multivessel lesions who were included in our study had STEMI, and there is currently no guidance for the staged revascularization strategy for multivessel lesions in non-STEMI patients. Finally, due to the fact that the duration of the included studies was mostly one year, this meta-analysis may not be sufficient for comparing long-term adverse events between immediate PCI and staged PCI.

## Conclusion

In summary, staged multivessel PCI significantly reduces the risk of myocardial infarction and unplanned ischemia-driven revascularization in randomized trial-based analyses. There was no significant difference between the two groups in terms of all-cause mortality, cardiovascular mortality, or noncardiovascular mortality risk. However, prospective non-randomized studies suggest there might be a benefit in mortality in the staged PCI group. In addition, there was no significant difference between the immediate PCI group and the staged PCI group in terms of ischemic stroke, stent thrombosis, renal function injury, or major adverse bleeding events. Therefore, staged multivessel PCI may be the optimal PCI strategy for STEMI patients with MVD.

## Data Availability

The original contributions presented in the study are included in the article/Supplementary Material, further inquiries can be directed to the corresponding authors.
