# Supplementary material for: The optimal timing for intervention in patients with ST-segment elevation myocardial infarction and multivessel disease: a systematic review and meta-analysis
Source: Front Cardiovasc Med. 2024 Aug 9;11:1389017. doi: 10.3389/fcvm.2024.1389017 (PMC11341378; doi:10.3389/fcvm.2024.1389017)

**Supporting Information**

**Supporting Information Table 1:**

| Study | Definition of MVD | Inclusion criteria | Exclusion criteria |
| --- | --- | --- | --- |
| Stahli 2023 (MULTISTARS AMI) | the presence of angiographically relevant stenosis (stenosis of ≥70% of the artery diameter on coronary angiography, as estimated on the basis of a visual assessment) in at least one nonculprit coronary artery that was at least 2.25 to 5.75 mm in diameter. | acute STEMI < 24 hours with MVD | (1)Inability to give informed consent; (2)Cardiogenic shock :Persistent systolic blood pressure below 90 mmHg or needed infusions of  catecholamines to maintain systolic blood pressure >90 mmHg and/or clinical signs of hypoperfusion; (3)Prolonged resuscitation >10 min; (4)General unsuitability for PCI ; (5)Need for emergency coronary artery bypass grafting (CABG); (6)Previous CABG; (7)Planned hybrid revascularization: Hybrid revascularization is defined as the intentional combination of PCI and CABG; (8)Coronary dissection |
| Park 2023 (COCUA) | NA | (1) age > 18 years; (2) acute STEMI < 24 hours with MVD (more than 2 significant target lesions in the different target native coronary arteries) | (1) adverse reactions to drugs such as hepar in, aspir in, clopidogrel, ticlopidine, everolimus, and contrast agent; (2) systemic (intravenous) everolimus use within 12 months; (3) pregnancy; (4) history of bleeding diathesis or known coagulopathy (including heparin-gastrointestinal or genitourinary bleeding within the prior 3 months, major surgery within 2 months, or induced thrombocytopenia); (5) planned elective surgical procedure that would necessitate thienopyridine inter r uption during the first 6 months after enrollment; (6) life expectancy < 1 year owing to a non-cardiac-related disease (per site investigator’s medical judgment); (7) actively participating in another drug or device investigational study which has not completed the primary end point follow-up period; and (8) left ventricular ejection fraction (LVEF) < 25% or presence of cardiogenic shock. |
| Diletti 2023 (BIOVASC) | two or more coronary arteries with a diameter of 2·5 mm or more and ≥70% stenosis based on visual estimation or positive coronary physiology testing | aged 18–85 years presenting with STEMI or non-ST-segment elevation acute coronary syndrome and multivessel coronary artery disease with a clearly identifiable culprit lesion | (1) previous coronary artery bypass surgery; (2) cardiogenic shock; (3) single-vessel coronary disease; (4) the presence of a coronary chronic total occlusion; (5) The absence of a clear culprit lesion |
| Kim 2021 | having an additional ≥70% diameter stenosis in at least one major non-IRA or in the left main coronary artery | patients with STEMI and MVD without cardiogenic shock in STEMI | (1) non-STEMI; (2) arrived >12 h after symptom onset; (3) cardiogenic shock; (4) thrombolysis before PCI; (5) single-vessel disease; (6) failed PCI for IRA; (7) lost to follow-up |
| Ahn 2020 (KAMIR-NIH) | NA | patients with STEMI and MVD | patients with culprit only P-PCI, multi-vessel partial revascularization, facilitated PCI or delayed PCI, defined as a PCI was not performed within 12 hours of ischemic symptom presentation, fibrinolytic therapy, conservative treatment or missing data |
| Kim 2017 | critical stenosis (>70% of diameter stenosis) in at least 2 major epicardial coronary arteries on a diagnostic coronary angiogram | patients with STEMI and MVD | (1) hemodynamic instability and history of coronary artery bypass grafting; (2) history of bleeding diathesis, conditions predisposing to hemorrhagic risk, or refusal to receive blood transfusions; (3) stroke or transient ischemic attack within 6 months; (4) recent or known platelet count <100000 cells/mm3 or hemoglobin <10 g/dL |
| Tarasov 2017 | diameter stenosis (≥70%) of two or more arteries and/or significant branches of coronary arteries (diameter ≥2.5 mm) | (1) Age ≥18 years old; (2) Written informed consent prior to revascularization; (3) Multivessel native coronary arteries significant stenosis and primary PCI ≤12h from STEMI manifestations and ST-segment elevation ≥1 mm in ≥2 limb electrocardiographic leads or precordial leads ≥2 mm ST-segment elevation; (4)Diameter of coronary artery ≥2.5 mm; (5) ≥30 minutes chest pain | (1) No MVCAD; (2) Cardiogenic shock; (3) Significant left main stenosis (≥50%); (4) Contraindication to use heparin,aspirin, clopidogrel, ticagrelor, zotarolimus |
| Chung 2016 | diameter stenosis of more than 70% estimated visually in two or more major epicardial coronary arteries, including the infarct-related artery | patients with STEMI and MVD | Patients with PCI that was unsuccessful, based on angiography, or with contraindication to the administration of aspirin, heparin or clopidogrel |
| Kornowski 2011 (HORIZONS-AMI) | NA | patients ＞18 years of age with symptom onset within 12h of duration and ST-segment elevation of ＞1 mm in ＞2 contiguous leads, new left bundle branch block, or true posterior MI | (1) contraindications to any of the study medications; (2) prior administration of fibrinolytic therapy, bivalirudin, GPI, low molecular weight heparin, or fondaparinux for the present admission (prior unfractionated heparin was allowed); (3) current use of warfarin; (4) history of bleeding diathesis, conditions predisposing to hemorrhagic risk, or refusal to receive blood transfusions; (5) stroke or transient ischemic attack within 6 months or any permanent neurologic deficit; (6) recent or known platelet count ＜100,000 cells/mm3 or hemoglobin ＜10 g/dl; (7) planned elective surgical procedure that would necessitate interruption of thienopyridines during the first 6 months after enrollment; (8) coronary stent implantation within 30 days; (9) noncardiac comorbid conditions with life expectancy ＜1 year |
| Maamoun 2011 | NA | ST-elevation MI presented within 12h from the onset of symptom who had at least two angiographically-documented diseased coronary arteries (luminaldiameter narrowing P70%) | (1) Patients with cardiogenic shock, pulmonary edema, and left main coronary artery disease were excluded; (2) serum creatinine level >1.4 mg/dl and contraindication for anti-platelet therapy |
| Politi 2010 | >70% diameter stenosis of two or more epicardial coronary arteries or their major branches by visual estimation | patients with the presence of prolonged (more than 30 minutes) chest pain, started less than 12 h before hospital arrival and ST elevation of at least 1 mm in two or more contiguous limb electrocardiographic leads or 2 mm in precordial leads | Patients with cardiogenic shock at presentation (systolic blood pressure #90 mm Hg despite drug therapy), left main coronary disease ($50% diameter stenosis), previous coronary artery bypass grafting (CABG) surgery, severe valvular heart disease and unsuccessful procedures |

**Supporting Information Figure 1: Risk assessment of bias in 5 RCTs.**


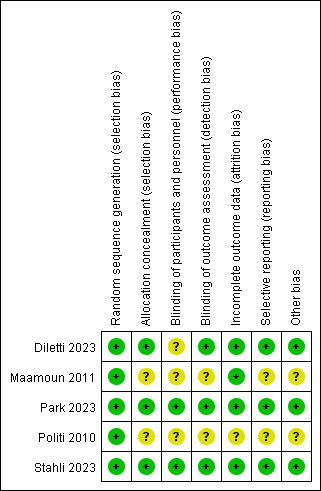

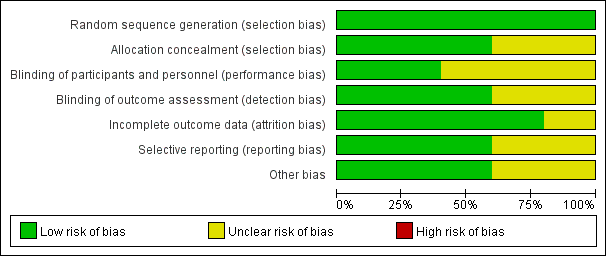


**Supporting Information Figure 2: The incidence of myocardial infarction and ischemia driven-revascularization based on real-world prospective studies.**


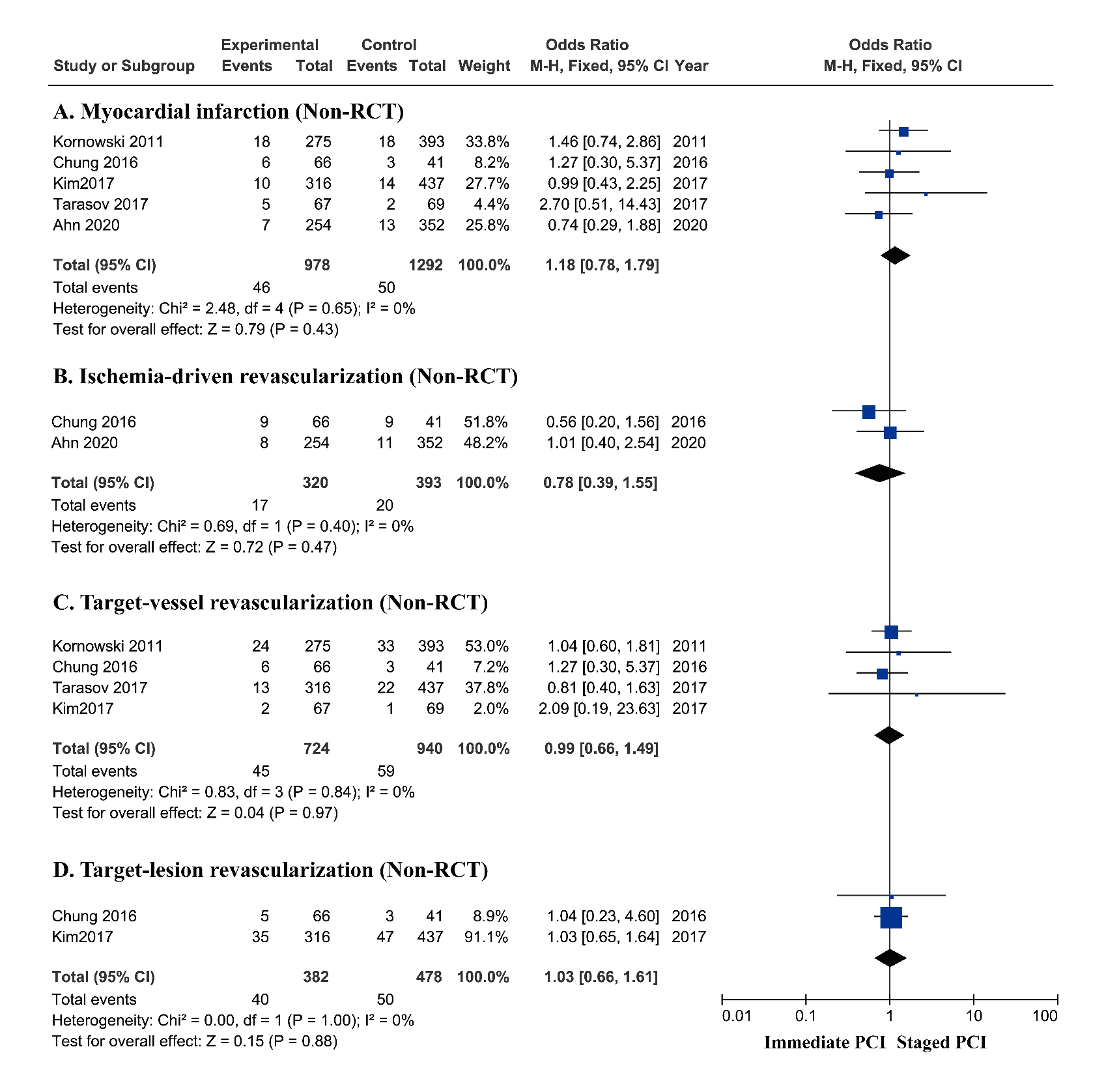


**Supporting Information Figure 3:** **Adverse outcomes of prospective studies based on the real world.**


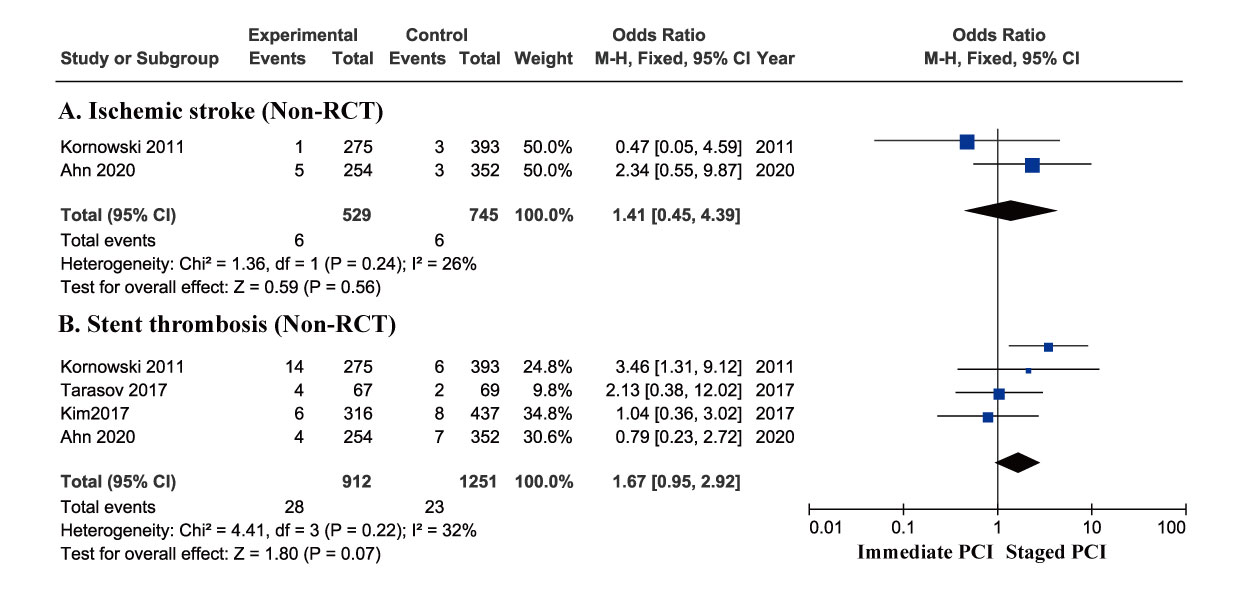

Supplement: Supplementary file 1 [file Datasheet1.docx]
